# Current Concepts in the Management of Necrotizing Fasciitis

**DOI:** 10.3389/fsurg.2014.00036

**Published:** 2014-09-29

**Authors:** Evangelos P. Misiakos, George Bagias, Paul Patapis, Dimitrios Sotiropoulos, Prodromos Kanavidis, Anastasios Machairas

**Affiliations:** ^1^3rd Department of Surgery, Attikon University Hospital, University of Athens School of Medicine, Athens, Greece

**Keywords:** necrotizing fasciitis, Fournier’s gangrene, gas gangrene, surgical debridement

## Abstract

Necrotizing fasciitis (NF) is a severe, rare, potentially lethal soft tissue infection that develops in the scrotum and perineum, the abdominal wall, or the extremities. The infection progresses rapidly, and septic shock may ensue; hence, the mortality rate is high (median mortality 32.2%). Prognosis becomes poorer in the presence of co-morbidities, such as diabetes mellitus, immunosuppression, chronic alcohol disease, chronic renal failure, and liver cirrhosis. NF is classified into four types, depending on microbiological findings. Most cases are polymicrobial, classed as type I. The clinical status of the patient varies from erythema, swelling, and tenderness in the early stage to skin ischemia with blisters and bullae in the advanced stage of infection. In its fulminant form, the patient is critically ill with signs and symptoms of severe septic shock and multiple organ dysfunction. The clinical condition is the most important clue for diagnosis. However, in equivocal cases, the diagnosis and severity of the infection can be secured with laboratory-based scoring systems, such as the laboratory risk indicator for necrotizing fasciitis score or Fournier’s gangrene severity index score, especially in regard to Fournier’s gangrene. Computed tomography or ultrasonography can be helpful, but definitive diagnosis is attained by exploratory surgery at the infected sites. Management of the infection begins with broad-spectrum antibiotics, but early and aggressive drainage and meticulous debridement constitute the mainstay of treatment. Postoperative management of the surgical wound is also important for the patient’s survival, along with proper nutrition. The vacuum-assisted closure system has proved to be helpful in wound management, with its combined benefits of continuous cleansing of the wound and the formation of granulation tissue.

## Introduction

The term necrotizing fasciitis (NF) describes a group of relatively uncommon, but life-threatening infections of the skin, soft tissues, and muscles, which tend to progress rapidly through the fascia planes, causing gradual destruction of the fascia at a rate reaching 2–3 cm/h. Developing in the lower or upper extremities, the perineum and genital area (Fournier’s gangrene) and in the abdominal wall, its swift clinical course is correlated with polymicrobial infection and synergy, which usually co-exists (1, 2). The majority of cases present anaerobic bacteria that proliferate in a hypoxic environment and produce gas, which accumulates in the soft tissue spaces, giving the characteristic image of gas gangrene on plain X-rays and computed tomography (CT) scans (3).

Early diagnosis of NF is mandatory. Any delay could prove fatal, given its association with more extensive surgery, higher rates of amputation, and higher mortality rates. Furthermore, if left untreated, the infection could lead to systemic inflammatory response syndrome (SIRS).

Necrotizing fasciitis was first recognized in 500 BC, when Hippocrates reported a clinical description of a complication of erysipelas disease, resembling the current description of NF (4). In France, Claude Colles, chief surgeon of the Hotel Dieu in Lyon, described a condition in 1783 that was very similar to modern descriptions of NF (5). The first description of “modern” NF was made by Joseph Jones, a military surgeon of the army of the Confederate States of America. In 1871, he reported 2,642 cases of gas gangrene treated in hospital during the American Civil War, with a mortality rate of approximately 46% (6). In 1883, Jean Alfred Fournier described a syndrome with necrosis of the perineum in five men; this type of NF was subsequently given his name and is known as *Fournier’s gangrene* (7). In 1924, Meleney reported an association with beta-hemolytic streptococcus A in a study of a series of hospitalized cases in Beijing. Thereafter, these cases were described for several decades as *Meleney’s gangrene* (8). In 1952, the term “*necrotizing fasciitis*” was proposed by Wilson, as a more accurate description of this disease (9). The late 1980s witnessed a renewed interest in this pathology. Stevens reported that, among 20 patients who presented with streptococcal shock, 11 were diagnosed as having NF. The disease was popularized by the media as “flesh-eating bacteria syndrome” (10).

### Epidemiology

The annual incidence of NF is estimated at 500–1,000 cases annually, and its prevalence globally has been reported to be 0.40 cases per 100,000 population (11). It is seen to have a predilection for men, with a male-to-female ratio of 3:1; this ratio is mainly correlated with the increased incidence of Fournier’s gangrene in men. The disease affects all age groups, although middle-aged and elderly patients (over 50 years of age) are more likely to be infected (1). The median mortality ratio of NF is a controversial issue. In their review of the literature, Goh et al. concluded that the median mortality ratio was 21.5% (12). However, its range in the literature is extensive, varying from 8.7 to 76% (13). In regard to NF of the extremities, the mortality rate is slightly lower than that recorded for abdominal and perineal infections (14, 15). Patients with Fournier’s gangrene that has not spread to the abdominal wall tend to have a better survival. As a general rule, without treatment, the mortality rate approaches 100%. Anaya et al. (16) have demonstrated that infection of the lower extremities is the most common site of NF (57, 8%), followed by the abdomen and the perineum. NF of the upper limbs is rare compared to that of the lower limbs (17).

### Etiology

Trauma is the most common identifiable etiology. The majority of patients have a history of minor or major traumas, generally involving external injuries and surgical wounds. Appendicitis with perforation, infection following the repair of an incarcerated hernia, perforated diverticulitis, necrotic cholecystitis, gastroduodenal perforation, small bowel perforation, and obstructive colon cancer with perforation rank among the most frequent causes of complicated intra-abdominal infections that can lead to NF. Notably, the incidence of NF resulting from a surgical wound in the chest wall is greater than that recorded from analogous wounds in the lower abdominal wall. Such cases present a high risk of osteomyelitis, which substantially increases the mortality of these patients.

Fournier’s gangrene is often the result of surgical wounds, skin abscess drainage, and pressure sores. It can also present as a complication of colorectal disease due to anorectal infection, ischiorectal abscesses, and colon perforations. Other causes include a possible urethral stricture and a trauma from an indwelling Foley catheter. In women, it has commonly been ascribed to Bartholin abscesses or vulval skin infections.

In Asia, consumption of raw or undercooked seafood or injury by fish fins can lead to NF (12). In this group of infections, bacteria such as *Vibrio* spp., *Aeromonas* spp., and *Shewanella* spp. are commonly involved and are usually known as “marine bacteria” (18).

### Co-morbidities and risk factors

The most frequent co-morbidity in patients with NF is diabetes mellitus. The prevalence of diabetes mellitus in patients with any type of NF ranges between 40 and 60% (12, 19). Other common co-morbidities include liver cirrhosis, chronic heart failure, obesity, alcohol abuse, immunodeficiency, systemic lupus erythematosus, Addison’s disease, pre-existing hypertension, and peripheral vascular disease (20, 21). A septic condition and hypotension at the time of admission are significant predicting factors for mortality and outcome. Chronic renal failure is another indisputable predisposing factor for higher mortality in patients with NF. Elevated serum creatinine, along with elevated blood urea, is also strongly associated with higher mortality rates (22).

The use of non-steroidal anti-inflammatory drugs (NSAIDs) or steroid drugs can suppress fever, thereby hampering the diagnosis of NF (8). Furthermore, Martinschek et al. have demonstrated that an increase of serum creatine kinase and lactate parameters, as well as a decrease of serum antithrombin III, proved by a low INR, are significant parameters for an unfavorable outcome, particularly in regard to Fournier’s gangrene (20). Other risk factors, such as systemic acidosis, low hematocrit, and albumin levels, are also strongly linked with a high mortality, while concomitant conditions increase the mortality rate further (23). Patients displaying accompanying diseases are usually characterized as critically ill and require prolonged intensive care. Diabetes, in particular, is a disease, which often combines many of the above co-morbidities, and is hence susceptible to the development of NF (24). However, the presence of diabetes mellitus has not been proven to affect mortality (25).

Advanced age constitutes another risk factor for higher incidence and mortality, although somewhat controversial. Large population-based studies have shown that advanced age is a strong, independent predictor of mortality (26). A study by Rea and Wyrick reported a mortality rate of 67% in patients over 50 years of age and 4% in patients under that age (27). Other studies have concurred that advanced age is a risk factor for higher mortality, but only when accompanied by other risk factors such as renal failure, or delayed surgical debridement (24). This combination is also associated with advanced disease and a more fulminant infection.

Whether or not patients with NF show a gender predilection with regard to mortality is also a topic of debate. Fournier’s gangrene shows male predominance with a reported rate of 96% (28). Czymek et al. found that mortality was significantly higher among females (50% F vs. 7.7% M) (29). However, studies involving a larger study population have concluded that there is no statistical correlation between female gender and increased mortality (30).

The extension and variability of infection are assumed to increase mortality. NF can affect an entire extremity within 24 h (31), but it can also show slow progression over a period of several weeks. In some patients, the disease remains dormant and unexpectedly spreads rapidly without any readily apparent reason (24). The factors that lead to the fulminant form of NF, with a potentially lethal outcome within 24 h, remain unidentified (32). Some studies indicate that the spread of gangrene does not relate to a poorer prognosis. Notwithstanding, the extension of gangrene to the abdominal wall has been reported to be directly related to increased mortality (33).

### Pathophysiology

Infection begins in the hypodermis or the superficial fascia, as the more superficial layers (dermis and epidermis) are not affected at the beginning (34). The synergistic action of the virulence factors of bacteria and the specific factors of the host are implicated in the development of NF. The extension of the infection and necrosis is facilitated by the synergy between the different bacteria and toxins and the enzymes they produce (35). An anaerobic environment promotes growth of anaerobic bacteria. Necrosis of the hypodermis and superficial fascia is directly related to bacterial enzymes that destroy the fascia and fat, and secondarily to vascular origin. Invasive bacteria cause thrombosis of the nutrient vessels, which are located in the hypodermis, leading to tissue ischemia aggravated by the presence of edema. Tissue ischemia promotes infectious dissemination leading to skin necrosis at a later stage. It also explains the intense pain phenomena that are usually observed, especially when the nerve branches are also affected. Such cases also display signs of regional hypoesthesia/anesthesia. The fascial and hypodermic necrotic spread is greater than the overlying skin changes. Lymphangitis and lymphadenopathy are rare due to thrombosis of the vessels. Gas formed by anaerobic bacteria may lead to crepitus.

### Microbiology

Recent studies have concluded that NF can be classified into four types, according to microbiological findings (35)(Table 1). The most common is type I, also known as the *polymicrobial type*. Accounting for 70–90% of cases, it typically affects patients with several co-morbidities, such as diabetes mellitus. Two or more pathogens are implicated in this infection (with an average of 4.4 species) and it is mostly found in the trunk and perineum.

**Table 1:** **Classification of responsible pathogens according to type of infection**.

Microbiological type	Pathogens	Site of infection	Co-morbidities
Type I (polymicrobial)	Obligate and facultative anaerobes	Trunk and perineum	Diabetes mellitus
Type II (monomicrobial)	Beta-hemolytic streptococcus A	Limbs	
Type III	*Clostridium* species	Limbs, trunk, and perineum	Trauma
	Gram-negative bacteria		Seafood consumption (for *Aeromonas*)
	*Vibrios* spp.		
	*Aeromonas hydrophila*	
Type IV	*Candida* spp.	Limbs, trunk, perineum	Immunosuppression
	Zygomycetes	

Type II, otherwise known as monomicrobial, is defined as infection with beta-hemolytic Streptococcus A (*Streptococcus pyogenes*). *S. pyogenes* is commonly found in young and healthy patients with NF of the extremities. Its pathogenesis is explained by the several virulence factors produced by this organism (36). In some cases, the infection can be associated with *Staphylococcus aureus*. *S. aureus* secretes toxins, which cause leukocyte destruction and tissue necrosis. Found in the fulminant forms of NF, *S. aureus* is not easy to manage, especially when the responsible pathogen is the methicillin-resistant *S. aureus* (MRSA), which is the case in 10–30% of all patients. Typically, these infections occur after small incisions, and appear to be highly correlated with the use of NSAIDs (37). Specifically observed in patients without serious co-morbidities, the infection is most often found in the limbs. The risk of toxic shock syndrome is increased in such cases, and the outcome is unfavorable.

Type III includes monomicrobial infections involving the *Clostridium* species or Gram-negative bacteria. *Clostridium* species are anaerobic bacteria that can be produced by external injuries (deep wound or crush injury causing local devascularization) or surgical wounds (intestinal and obstetric). *Clostridium* infections are currently more frequent among drug addicts (38), and *C. perfringens* is the most common bacterium of the *C*. species. *Vibrios* spp. infections can also lead to type III NF. *Vibrio vulnificus* is a marine bacterium frequently isolated in Asia (21). *Aeromonas hydrophila* is found in freshwater or low salinity water and in the soil. The clinical symptoms of infections by these two bacteria are similar; hemorrhagic blisters, lesions, and purpuric necrosis are the dominant symptoms, along with the extremely rapid spread of disease.

Finally, type IV is the result of fungal infections, mainly *Candida* spp. and Zygomycetes. This type is found mainly in the immunocompromised host. Infections by these fungi often occur after trauma; the clinical image is aggressive and rapidly extensive, particularly in immunocompromised patients.

Microbiological diagnosis is obtained in almost 75% of all cases of NF (39), and is based on the good quality of the pre- and intraoperative samples and blood cultures. Blood cultures are positive in 25% of all cases, while cultures obtained from the site of injury during surgical debridement result as positive in 80% of cases (40).

## Diagnosis

### Clinical signs and symptoms

Patients with NF usually present with the classic triad of symptoms: local pain, swelling, and erythema (12). Tachycardia (>100 beats/min) and fever are the most common vital sign abnormalities, followed by hypotension (SAP < 100 mmHg) and tachypnea (>20/min). These vital sign abnormalities, along with the skin erythema, are most useful in securing the diagnosis of NF from other soft tissue infections (41). The infected site displays tenderness, sclerosis, skin necrosis, and hemorrhagic bullae (42).

Depending on the development of the infection, the clinical image described above may not always be evident. Consequently, two groups of symptoms are considered, namely early and advanced symptoms (43). The most common early signs are erythema, local warmth, skin sclerosis, and edema. However, in the fulminant form of disease, the patient is critically ill with signs and symptoms of severe septic shock and multiple organ dysfunction syndrome, along with extensive necrosis of soft tissue. In this case, the clinical picture deteriorates rapidly within a few hours; pain is severe and usually manifests before the cutaneous signs. Remarkably, pain seems to be disproportionate to the clinical findings.

In contrast, the subacute form of the disease has a relatively slow clinical course, which may endure for days or weeks. The early clinical status of the subacute form is the result of an existing condition leading to infection. The patient often presents with a skin infection, such as folliculitis or abscess, gangrene on the extremities, pressure sore(s), or a complicated surgical wound. Erythema or skin sclerosis is present at the site of infection. The patient usually feels pain at the site of the injury, and this is a very strong diagnostic hint. However, local nerves can also be infected, usually resulting in the partial loss of sensation (44).

As the infection develops, the pain becomes more intense. The clinical image is characterized by symptoms of general toxicity including fever, dehydration, confusion, dizziness, diarrhea, nausea, vomiting, weakness, and malaise (19). If the patient remains undiagnosed or untreated, the clinical status deteriorates rapidly. The cutaneous symptoms may progress to blisters and bullae, ultimately leading to circumscribed necrosis of the skin. Initially, the bullae contain serous fluid, but, as the infection progresses, they may become hemorrhagic. Gas formation can lead to crepitus in the overlying skin, indicating anaerobic infection, such as *C. perfringens*. This classical skin condition does not normally present until day five or later (45).

Symptoms of septic shock or MODS frequently appear in the late phase of its subacute form. As a result, the patient displays hypotension, elevated white blood cell count, metabolic acidosis, coagulopathy, changes in mental status, and weakness. In this late stage of the disease, the patient looks apathetic and indifferent. Additional symptoms pertaining to co-morbidities may also coexist.

The symptoms of disease are not characteristic; hence, it is often misdiagnosed as cellulitis or abscess. The most consistent feature of early NF is pain, which is not in proportion to the swelling or erythema (46). Moreover, as a consequence of the enzymatic and toxin action, tenderness to palpation extends beyond the area of apparent involvement, to spread along fascial plains. In addition, the margins of involvement are usually indistinct, and lymphangitis is rarely present, given that the infection is in the deep fascia rather than the skin (47).

Cases with upper limb infection do not always present a typical picture. In this instance, patients may appear systemically well (21). Another cause that complicates diagnosis is the absence of fever in most cases. Several drugs, such as NSAIDs, steroids, and antibiotics can lower body temperature and mask fever. For that reason, the absence of pyrexia does not necessarily exclude NF (12, 41).

Fournier’s gangrene has a slightly different clinical course. It usually begins with pain and itching of the perineum and scrotal skin. In genitourologic types of Fournier’s gangrene, the pathogens pass through the Buck’s fascia of the penis and spread along the Darto’s fascia of the scrotum and penis, Colle’s fascia of the perineum, and Scarpa’s fascia of the lower abdominal wall (19). Additional necrosis of the superficial fascia and fat produces a thin watery malodorous fluid and crepitus. Similarly, patients may present high fever, anxiety, altered mental status, leukocytosis, shock, and tachypnea, when shock is about to develop. Once clinical signs become obvious, the appearance resembles the late phase of NF, with visible bruising, bullae and cutaneous necrosis due to the extension of the necrotizing process.

### Bedside and laboratory tests

Laboratory results in this disease are not usually specific. However, certain laboratory findings can help the clinician to differentiate NF from other skin diseases, such as necrotizing soft tissue infection. Specifically, leukocytosis is a common feature in patients with NF (48), and white blood cell count in excess of 20,000/L is highly suspect. Blood urea nitrogen >18 mg/dL and serum creatinine >1.2 mg/dL reflect ongoing renal failure, which is typically present in these patients. Serum creatine kinase is also elevated (CK) in patients with severe sepsis and MODS (48). Majeski et al. suggested that C-reactive protein >16 mg/dL or creatine kinase >600 IU/L generally precludes group A β-hemolytic streptococcal infection (49). However, this recommendation is rarely followed by clinicians.

Several laboratory-based scoring systems have been proposed for establishing early diagnosis of NF (50). The Laboratory Risk Indicator for Necrotizing Fasciitis (LRINEC) proposed by Wong et al. (51–53) is one such example (Table 2). In addition to enabling early recognition of the disease, this score can also facilitate the classification of patients into risk categories, and help in the allocation of diagnostic resources. Another scoring system is the Fournier’s Gangrene Severity Index (FGSI) that has shown remarkable success in helping to determine whether or not the patient requires surgical debridement (54).

**Table 2:** **LRINEC scoring system for necrotizing fasciitis**.

Variable	Score
CRP (mg/L)
>150	4
WBC (g/L)
<15	0
15–25	1
>25	2
Hemoglobin (g/dL)
>13.5	0
11–13.5	1
<11	2
Sodium (mmol/L)
<135	2
Creatinine (μmol/L)
>141	2
Glucose (mmol/L)
<10	1

Bedside tests, imaging tests [CT or magnetic resonance imaging (MRI)], or frozen section biopsy can be carried out in patients with equivocal clinical findings and a moderate or high risk of NF based on the LRINEC score (>5). The finger test and frozen section biopsy are used as complementary diagnostic modalities in patients with an equivocal diagnosis. Surgical exploration is regarded as the mainstay for investigation and treatment. The *finger test* is a bedside procedure performed under local anesthesia by which, a 2-cm incision is made down to the deep fascia, at which level gentle probing of the index finger is applied. The presence of characteristic “dishwater pus,” along with the lack of bleeding and lack of tissue resistance to blunt finger dissection are positive findings correlated with NF. Another useful bedside test is an incisional biopsy down to the fascial level with an immediate frozen section, culture, and Gram stain (55, 56).

The combination of surgical exploration and microbiological and histopathological analysis of 1 cm^3^ of soft tissue is considered the gold standard for confirming diagnosis, when the latter is ambivalent.

### Imaging tests

Imaging investigation can help to establish the diagnosis of NF especially in equivocal cases. Although plain radiography has low sensitivity and specificity, it is capable of showing gas formation in the soft tissue (33), which is present in almost half of all patients, and it strongly indicates infection by the *Clostridium* species. CT and MRI are more sensitive and specific than plain radiography. A CT scan can show the extent of tissue infection, fascial swelling, inflammation, and gas formation. An MRI scan provides better accuracy than CT, though not widely used, due to cost. Ultrasonography is also a feasible option, providing useful information concerning the nature and extent of infection, especially when the diagnosis is unclear (57). In terms of diagnosis, the most significant finding is the hyperechoic foci with reverberation artifact and dirty shadowing at the site of infection (57), representing the subcutaneous gas. However, ultrasonography requires a highly skilled operator; this requirement hampers its use in the ICU, where patients with NF are usually treated.

## Treatment

### Antibiotic treatment

Since ischemia and hypoxia compromise the adequate delivery of antibiotics to the infection site, conservative treatment with antibiotics alone has little value in the management of NF (58). However, they play a significant role in surgical management of the infection. Patients should be immediately treated with broad-spectrum antibiotics, when NF is suspected. The empirical usage of antibiotics is based on the microbiological classification of NF. Antibiotic treatment of a polymicrobial infection should be based on history, Gram stain, and culture. Initial treatment includes ampicillin or ampicillin–sulbactam combined with metronidazole or clindamycin (59). Anaerobic coverage is quite important for type 1 infection; metronidazole, clindamycin, or carbapenems (imipenem) are effective antimicrobials. Broad gram-negative coverage is necessary as an initial empirical therapy for patients who have recently been treated with antibiotics, or been hospitalized. In such cases, antibiotics such as ampicillin–sulbactam, piperacillin–tazobactam, ticarcillin–clavulanate acid, third or fourth generation cephalosporins, or carbapenems are used, and at a higher dosage.

Type 2 disease is treated with antibiotics against *S. pyogenes* and *S. aureus*, which usually coexist with the former. Hence, first or second generation of cephalosporins are used for the coverage of methicillin-sensitive *Staphylococcus aureus* (MSSA). MRSA tends to be covered by vancomycin, or daptomycin and linezolid in cases where *S. aureus* is resistant to vancomycin. Some studies suggest that clindamycin is superior to penicillin in managing streptococcal infections (60), but this has yet to be satisfactorily proven. Another study has proposed that clinicians should consider adding clindamycin to the beta-lactam antibiotic regimen when NF or myositis is present (61).

Type 3 NF should be managed with clindamycin and penicillin, which cover the *Clostridium* species. If Vibrio infection is suspected, the early use of tetracyclines (including doxycycline and minocycline) and third-generation cephalosporins is crucial for the survival of the patient, since these antibiotics have been shown to reduce the mortality rate drastically (59).

Finally, type 4 NF can be treated with amphotericin B or fluoroconazoles, but the results of this treatment are generally disappointing.

As in every empirical antibiotic therapy, the dosage should be tapered, based on the results of the initial blood, wound, and tissue cultures, but continued until the infection is under control and for at least 48 h after clinical and hemodynamic stabilization of the patient has been achieved. Antibiotics should be administered for up to 5 days after local signs and symptoms have resolved (62). The mean duration of antibiotic therapy for NF is 4–6 weeks.

Intravenous immunoglobulin (IVIG) has recently been described as a reasonable and desirable option for neutralizing streptococcal toxins (63). There is evidence that a high dose of IVIG may prove beneficial in severe streptococcal infections (64), but this has yet to be demonstrated with randomized studies.

### Surgical management

Emergency surgical debridement of the affected tissues is the primary management modality for NF. Surgical debridement, necrosectomy, and fasciotomy are the main aspects of surgical treatment. Surgical intervention is life-saving and must be performed as early as possible, since a delay in treatment beyond 12 h in fulminant forms of NF can prove fatal. Surgical debridement should be repeated during the next 24 h or later, depending on the clinical course of the necrotizing infection and vital functions. Many studies have pointed out that timing and the extent of the first debridement are the most important risk factors in terms of increased mortality rate. Mock et al. have shown that the relative risk of death was 7.5 times greater in cases of restricted primary debridement (65), whereas, other studies reported that the mortality rate was nine times greater when primary surgery was performed 24 h after the onset of symptoms (19).

**Figure 1:**
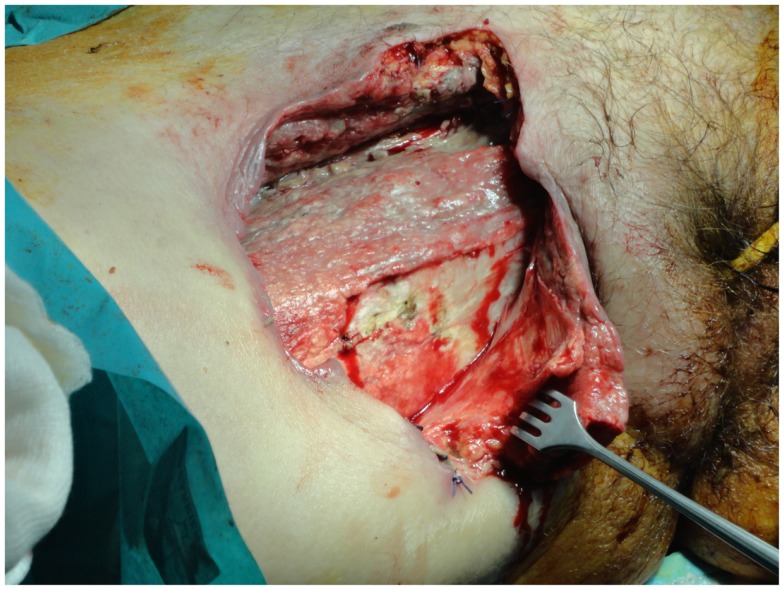
**The excision of the necrotic tissues should extend until healthy tissue is found, but should be limited to the edges of the infection**.

Surgical management is indicated especially for patients displaying intense pain and skin color change, such as edema and/or ecchymoses, or signs of skin ischemia with blisters and bullae (Figure 1). Patients must be operated on urgently when they present with altered mental status, hypotension, an elevated band formed in the differential WBC count, and metabolic acidosis. These clinical and laboratory signs indicate that the patient has developed SIRS or MODS, and the NF score has risen to phase 3. The mortality rate in this phase is extremely high, reaching almost 70% (24).

Incisions are performed parallel to Langer’s lines to achieve better surgical wound healing and less scarring. Surgery also minimizes the overall tissue loss as it inhibits infection spread to the fascial plane, reducing the need for amputation (66). After the release of pus and/or hemorrhagic fluid through incisions, ventricle incisions are made, keeping the wound open in order to allow drainage and to remove additional necrotic tissue.

Patients with NF should be closely monitored during the next 24 h; surgical wounds and tissue viability should be checked. Complicated surgical wounds command a “second-look operation” with radical surgical debridement. Patients with NF can require from 5 up to 40 additional operations, depending on the timing of the first surgical debridement, the adequacy of the primary debridement and necrosectomy, signs of hemodynamic instability, and concomitant illnesses, all of which are associated with a high mortality rate (67). Evidence of hemodynamic instability demands immediate resuscitation, transfer to an intensive care unit, nutritional support, and enteral feeding.

The extent of tissue extracted depends on the body region, which is infected. As a general rule, debridement will extend until healthy tissue is found, though some authors recommend that excision should be limited to the edges of infection (68). The general consensus is that careful trimming of the potentially salvageable soft tissue is also required (while non-infected skin remains unattached) (Figure 1) (69).

Nutritional support is required from the first day of the patient’s admission to hospital (preferably the ICU), to replace lost proteins and fluid from large wounds and/or the resultant toxic shock. Metabolic demands are similar to those of other major trauma or burns, which means that the patient needs twice the basic caloric requirements.

Necrotizing fasciitis of the abdominal wall requires special consideration. Skin incision must be performed in the longitudinal direction along the muscle-fascial layers of the inner abdominal wall until healthy fascia is found. Parallel or ventricle incisions are not performed because the bridges of skin and skin islands will not usually survive. Postoperative management of abdominal wall wounds involves serial dressing changes over the following days, until the wound is free of recurrent or ongoing infection. The use of a vacuum-assisted wound closing device (VAC) can also be helpful. Aggressive surgical debridement should be repeated in cases of infection progression across the deep fascial planes of the abdominal wall. The extension of infection into the bowel, resulting in bowel ischemia, bowel obstruction, and peritonitis, is not an uncommon phenomenon. In such cases, an exploratory laparotomy is needed to estimate the extent of infection inside the abdominal wall. A radical surgical debridement at the site of infection and the retroperitoneal site is performed, followed by partial bowel excision, depending on the part of the bowel (usually right colon), which has been infected. A diverting colostomy is performed with multiple drainages of the infected intra-abdominal fluid collections. Surgical management of colonic perforation complicated with peritonitis is a topic with considerable debate in the literature. Hartmann’s resection has been considered the procedure of choice in cases with diffuse peritonitis and remains a safe technique for colectomy in a perforated colon, especially in elderly patients with multiple co-morbidities. As concerns NF, Hartmann’s resection is particularly preferred, since it allows time for reconstruction of the abdominal wall defects, and the diverting colostomy can be closed at a second stage. The primary defect on the abdominal wall is usually large and is repaired with advanced flaps using an abdominoplasty technique, biological mesh, or skin grafts (70).

**Figure 2:**
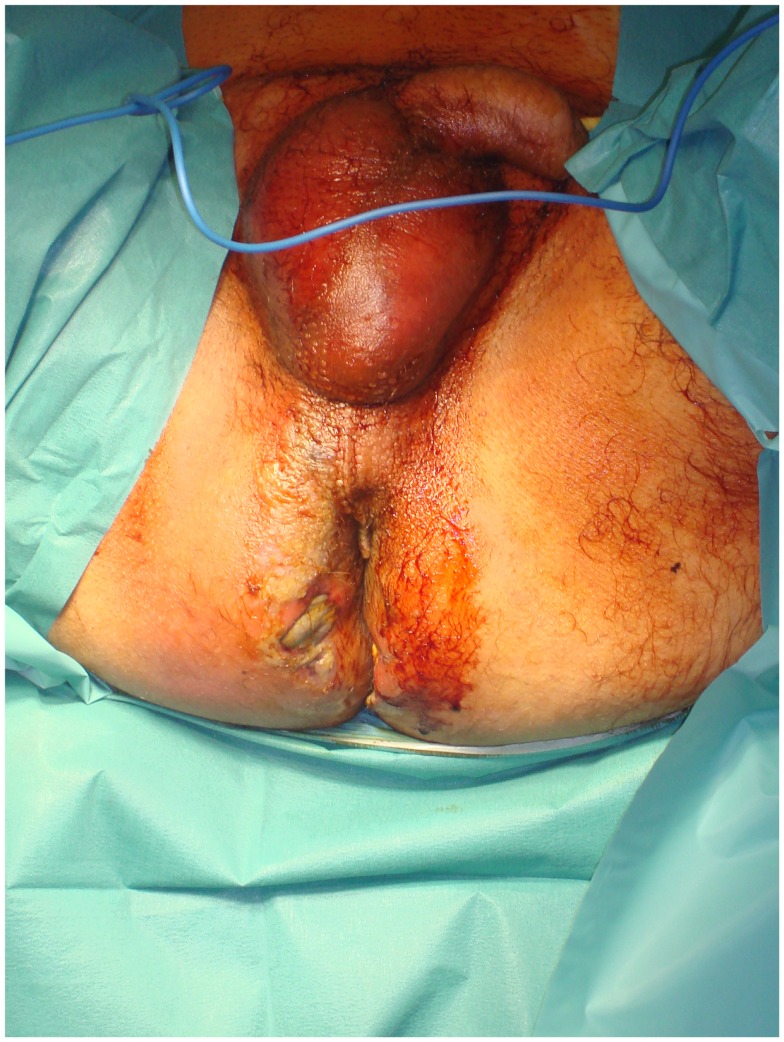
**A severe case of Fournier’s gangrene with excessive erythema and edema in the perineal and gluteal regions as well as skin necrosis with bullae**.

Fournier’s gangrene also requires special consideration. A pressure sore, perineal abscess, or paraplegia frequently predispose to the spread of infection into the scrotum, inguinal region, and lower abdominal wall (Figure 2). An orchiectomy, cystostomy, or diverting colostomy is often required dependent on whether the infection has extended to the scrotum, perineal area, or lower abdominal wall, respectively. Surgical management includes wide tissue incision, radical debridement, and drainage of the areas involved (71). The wound is washed with hydrogen peroxide, saline, and 1% povidone iodine solution. Finally, it is covered with occlusive and adsorptive dressing with antiseptic properties. Again the use of VAC can accelerate the recovery period, providing clean surgical wounds. Once the patient is clinically and hemodynamically stable, they can be submitted to reconstructive surgery.

Necrotizing fasciitis of the extremities needs special consideration. The extent of debridement is very important as additional fasciotomies are needed in cases with compromised tissue viability (67). The amount of tissue that needs to be excised is a controversial issue, because the skin in the extremities usually appears normal.

A study by Andreasen et al. showed that despite a normal external appearance soft tissue in patients with NF has extensive vascular microthromboses as well as vasculitis. The risk of full-thickness necrosis is high, and this can complicate a primary treated surgical wound (72). Consequently, it is recommended that clinicians also remove healthy soft tissue, bearing in mind that extremities showing NF may require amputation. The criteria for amputation have been recommended by Tang et al. (73), the most significant of which is extensive soft tissue necrosis with involvement of the underlying muscles and rapidly progressing infection with a large area of tissue necrosis. Other conditions that may justify amputation, are the presence of concurrent medical disease with high anesthetic risk (ASA score III and above), and the presence of shock (toxic or cardiogenic) requiring treatment with more than one inotrope. Furthermore, concurrent vascular insufficiency further increases the need for amputation, especially when the patient is diabetic. Amputation is usually considered as a shorter procedure associated with less blood loss than a radical debridement. This explains why patients with hypotension and shock are best treated with amputation, as they cannot endure additional protracted operations. Studies have proved that, although amputation is not seen to reduce mortality, patients undergoing this procedure required fewer repeat operations, which is extremely important for patients presenting severe co-morbidities or a fulminant form of NF (74).

Recent reports of NF of the axilla require special consideration. A delay in surgical debridement can prove lethal, even more so than an infection in a common site (75). Tissue in the axillary region that is rich in blood vessels and lymphatics enables the infection to spread rapidly to distant sites. The clinician should keep in mind the need to avoid axillary contractures, after covering the exposed neurovascular network (75). Again, surgical reconstruction of the wound is essential for successful wound closure and should be planned after clinical stabilization (76, 77).

### Use of vacuum-assisted closure device

**Figure 3:**
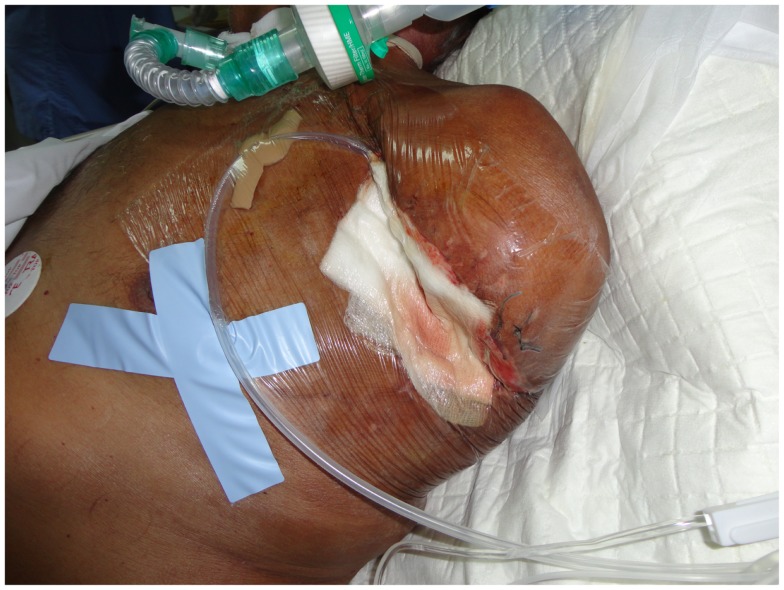
**After surgical debridement, the use of the VAC system helps wound healing by absorbing excess exudates; reducing localized edema, and finally drawing wound edges together**.

Lately, many surgeons worldwide have started using vacuum-assisted closure (VAC) therapy for fast and effective wound closure (78). Several studies in the general surgery, orthopedic, and gynecological literature support the use of VAC devices. A VAC device consists of a sterile, open-cell foam sponge that is placed in the wound, the size of which is adjusted to the wound size. This is covered with a transparent adhesive drape to create an airtight environment. The sponge is connected to a portable vacuum pump by means of non-collapsible tubing. Evacuation is applied to the sponge using the pump, which provides continuous negative pressure. The VAC device improves wound healing by providing microstrain (Figure 3). Several randomized studies have demonstrated improved wound healing and a significant reduction of wound surface area in full-thickness wounds treated with VAC devices as compared to conventional gauze therapy (79).

The VAC dressing must be changed every 24–72 h. VAC therapy has several benefits in wound management, with wound area reduction and formation of granulation tissue being the most prominent. Other benefits, such as effective wound cleaning and the ability to remove the exudate render VAC a promising adjuvant therapy for wound closure.

### Future therapies

As a life-threatening condition, NF demands new management strategies. Unfortunately, there are no single new therapies that can manage NF; they all seem to play an assistive role. Undoubtedly, the use of VAC has many benefits in wound healing, and it will be adopted by more physicians in the future.

A study by Anaya et al. (13) highlighted the role of IVIG in the treatment of NSTI, especially if NSTI is associated with group A streptococcal infection. The authors concluded that the use of IVIG seemed rational in patients with group A streptococcal infection who developed streptococcal toxic shock syndrome and in those with a high mortality risk (advanced age, hypotension, and bacteremia). However, relevant studies investigating its use are contentious and difficult to compare because of the small number of patients and the different methodologies used.

An interesting study by Lu et al. (80) showed that kallistatin, originally found to be a tissue kallikrein-binding protein, can increase the survival of group A streptococcus infected mice. The researchers concluded that kallistatin significantly increased the survival rate of GAS-infected mice, and also reduced local skin damage and bacterial counts. Moreover, its use improved infiltrating cell viability in the local infection site, as well as bacterial clearance activity of immune cells (81). The efficiency of intracellular bacterial killing in neutrophils was directly enhanced by kallistatin administration. Several inflammatory cytokines, including tumor necrosis factor alpha, interleukin 1β, and interleukin 6, in local infection sites were reduced by kallistatin. Furthermore, kallistatin treatment was reported to reduce vessel leakage, bacteremia, and liver pathology after local infection. However, further studies are warranted before safe conclusions can be drawn concerning its use in gas-forming infections, such as NF (82).

## Conclusion

Necrotizing fasciitis is a rare but life-threatening condition, with a high mortality rate (median mortality 32.2%) that approaches 100% without treatment. Numerous conditions are associated with this pathology, such as diabetes mellitus, immunosuppression, chronic alcohol disease, chronic renal failure, and liver cirrhosis, which can be conductive to the rapid spread of necrosis, and increase in the mortality rate. The diagnosis of NF is difficult and the differential diagnosis between NF and other necrotizing soft tissue infections more so. However, the clinician should do their utmost to secure the diagnosis of NF, as a delay in diagnosis can be fatal, and septic shock is inevitable if the disease remains untreated. The characteristic of NF is the clinical status change over time. The early clinical picture includes erythema, swelling, tenderness to palpation, and local warmth; once the infection develops, the infection site presents skin ischemia with blisters and bullae. The diagnosis of NF can be secured faster with the use of laboratory-based scoring systems, such as the LRINEC score or the FGSI score, especially in cases of Fournier’s gangrene. However, the diagnosis is definitely established by performing explorative surgery at the infected site.

Management of the infection begins with antibiotic treatment. In the majority of cases with NF (70–90%) the reasonable pathogens are two or more, suggesting the use of broad-spectrum antibiotics. The value of antibiotic treatment in NF is relatively low, and early and aggressive drainage and debridement is required. In NF of the extremities, the clinician should consider amputating the infected limb, although this will not reduce the risk of mortality. Finally, postoperative management of the surgical wound is important, along with proper nutrition of the patient. The use of VAC therapy in wound management has greatly improved the results of postoperative management.

## Conflict of Interest Statement

The authors declare that the research was conducted in the absence of any commercial or financial relationships that could be construed as a potential conflict of interest.
